# Iron Deficiency, Cadmium Levels, and Kidney Transplant Outcomes in Prevalent Kidney Transplant Recipients

**DOI:** 10.1016/j.xkme.2024.100942

**Published:** 2024-12-20

**Authors:** Pien Rawee, Daan Kremer, Ilja M. Nolte, Mark R. Hanudel, Daan J. Touw, Martin H. De Borst, Stephan J.L. Bakker, Michele F. Eisenga

**Affiliations:** 1 Department of Internal Medicine, Division of Nephrology, University of Groningen, University Medical Center Groningen, Groningen, The Netherlands; 2 Department of Epidemiology, University of Groningen, University Medical Center Groningen, Groningen, The Netherlands; 3 Department of Pediatrics, David Geffen School of Medicine, University of California Los Angeles, Los Angeles; 4 Department of Clinical Pharmacy and Pharmacology, University of Groningen, University Medical Center Groningen, Groningen, The Netherlands

To the Editor:

Cadmium is a nephrotoxic heavy metal. Previously, we showed that elevated plasma levels of cadmium are independently associated with an increased graft failure risk in kidney transplant recipients (KTRs).^1^ It appears that even at low levels, long-term cadmium exposure in KTRs with co-occurring oxidative stress from other factors can already be nephrotoxic.^1^^,^^2^ Besides the exposure level, an important contributor to cadmium levels in KTRs could be iron deficiency, which is highly prevalent in this patient setting.^3^ During iron deficiency, the divalent metal transporter 1 (DMT1), the central transporter for intestinal ferrous iron absorption, is being upregulated.^4^^,^^5^ A similar process might occur in the human kidney, as DMT1 upregulation has been observed in the kidneys of iron-deficient rats.^6^ Interestingly, DMT1 also transports cadmium with high affinity^7^ and iron deficiency is associated with increased levels of cadmium in several other populations.^8^ Therefore, the aim of this study was to elucidate whether iron deficiency is associated with increased cadmium levels in KTRs and whether the association of plasma cadmium with graft failure depends on iron status.

We used data from KTRs ≥1 year after transplantation who participated in the TransplantLines Food and Nutrition Cohort study. Linear regression analyses were applied to study the association between transferrin saturation (TSAT) and ferritin with log-transformed plasma cadmium while adjusting for age, sex (a person's biological characteristics, male or female), body mass index, and estimated glomerular filtration rate (eGFR). Correction for eGFR is performed because reduced eGFR might be both a consequence and cause of increased cadmium levels.^9^ Cox regression analyses were used to investigate whether the association of plasma cadmium with graft failure differed depending on TSAT and ferritin levels. Models were adjusted for age, sex, smoking behavior, body mass index, eGFR, 24-hour urinary albumin excretion, transplant vintage, human leukocyte antigen mismatches, donor type, and clinical history of acute rejection. A further description of the methods can be found in Item S1.

We included 574 stable KTRs (mean age 53 ± 12 years, 58% males, and mean eGFR 52 ± 20 mL/min/1.73 m^2^) at a median of 5.3 (interquartile range [IQR] 1.9-11.4) years after transplantation at baseline (Table S1). Plasma cadmium levels were significantly higher (*P* = 0.02, Fig 1A) in KTRs in the lowest TSAT tertile (≤20% [n = 192], median cadmium 6.2 [IQR 4.9-7.7] ng/dL), compared with KTRs in the highest tertile (≥29% [n = 191], median cadmium 5.6 [IQR 4.4-7.2] ng/dL). Linear regression analysis revealed that TSAT levels <20% were continuously inversely associated with log-transformed plasma cadmium concentrations, independent of potential confounders (β = –0.29, *P* = 0.02, Fig 1B). No association between ferritin levels and cadmium levels was observed (*P* = 0.91).

**Figure 1:**
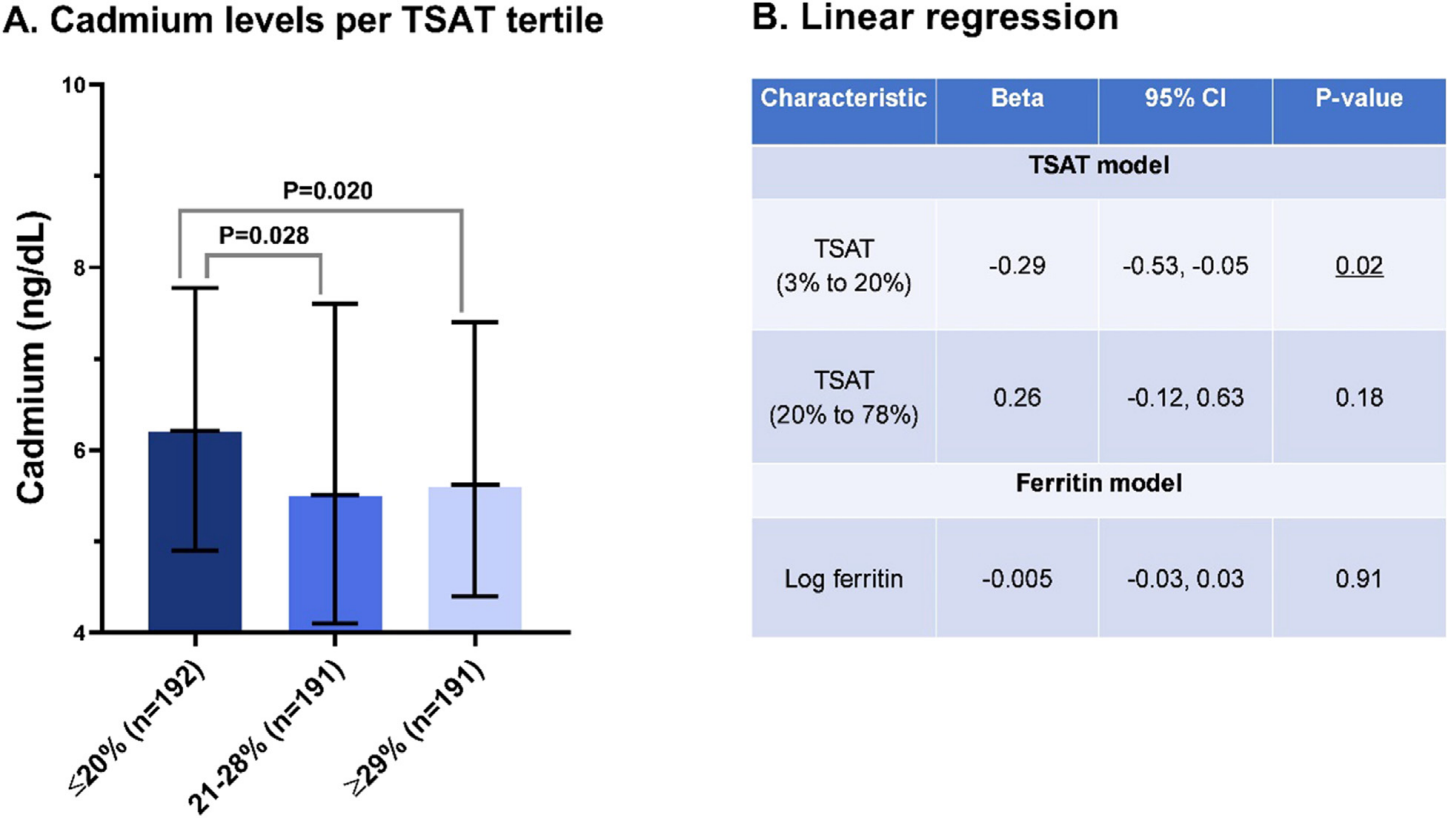
The interplay between iron status and plasma cadmium in kidney transplant recipients. (A) Wilcoxon rank sum tests were used to compare plasma cadmium concentrations between tertiles of TSAT. The black lines depict the median and interquartile range. (B) Linear regression with log-transformed plasma cadmium concentrations as the outcome and iron parameters as the predictors. The models are corrected for age, sex, smoking behavior, body mass index, and estimated glomerular filtration rate. Visual inspection of the relationship between iron status parameters and log-transformed cadmium showed an opposing association of TSAT with log cadmium around the iron deficiency cut-off value of TSAT 20%. Therefore, the linear regression was estimated using natural splines with a knot placed at TSAT 20%. Abbreviations: CI, confidence interval; TSAT, transferrin saturation.

During a median follow-up time of 5.6 (IQR 5.3-6.4) years, 69 KTRs developed graft failure (Fig S1). Cadmium was significantly and independently associated with the development of graft failure (hazard ratio [HR] 1 ng/dL increase 1.09; 95% CI 1.02-1.17, *P* = 0.02). Furthermore, we identified a significant positive interaction between the lowest tertile of TSAT (≤20% [n = 192] and cadmium on graft failure [HR 1.21; 95% CI 1.01-1.44; *P*_*interaction*_ = 0.04], Table S2). To visualize the latter, the association of cadmium with graft failure above and below the common iron deficiency cut-off of TSAT 20% is shown in Figure 2. No interactive effect between the lowest ferritin tertile with cadmium on graft failure was found (*P*_*interaction*_ = 0.87).

**Figure 2:**
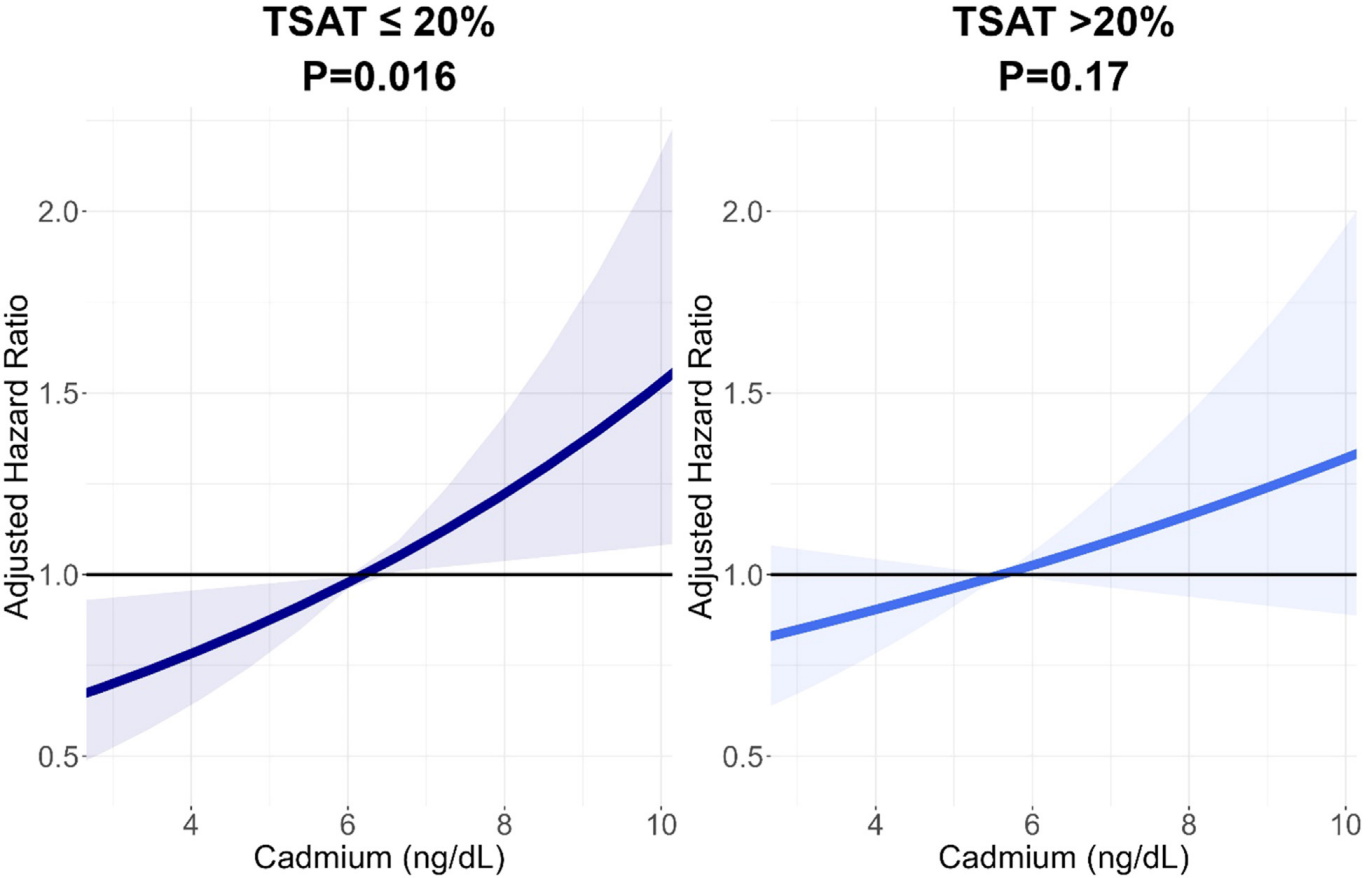
The association between plasma cadmium concentrations and graft failure in kidney transplant recipients with and without iron deficiency. Models were fitted and plotted in kidney transplant recipients with iron deficiency (TSAT **≤**20%, N *=* 192) and without iron deficiency (TSAT >20%, N *=* 382). The hazard ratio on the y-axis is adjusted for age, sex, smoking behavior, body mass index, estimated glomerular filtration rate, 24-hour urinary albumin excretion, time since transplantation, human leukocyte antigen mismatches, donor type, and clinical history of acute rejection. Abbreviation: TSAT, transferrin saturation.

In this study, we show that KTRs with TSAT ≤20% have higher plasma cadmium concentrations than KTRs with higher TSAT levels. In addition, within the TSAT range <20%, TSAT was significantly inversely associated with plasma cadmium concentrations. Finally, we identified an interactive effect between low TSAT and cadmium on graft failure.

Our results are in line with studies from other populations in which iron status was predictive of cadmium levels.^8^ Mechanistically, it is well established that DMT1 in the gut, which is able to transport both iron and cadmium, is being upregulated during iron deficiency.^4^^,^^5^ This likely explains the increased plasma cadmium concentrations of KTRs with TSAT <20% (ie, iron deficiency) in the current study. Interestingly, ferritin, was not associated with plasma cadmium concentrations. Ferritin is also an acute-phase protein and in fact, TSAT appears more reliable in reflecting iron status in patient groups with ongoing inflammation (eg, KTRs).^10^ We hypothesize that the interactive effect between low TSAT and cadmium on graft failure is due to an increased cadmium uptake in the kidney, as DMT1 upregulation has been observed in the kidneys of iron-deficient rats,^6^ but the latter should be further evaluated.

The strength of the current study is the large cohort, plasma cadmium availability, iron status, and several potential confounders. Given the study design, no causality can be attributed. Baseline characteristics significantly different across TSAT tertiles were included in the Cox regression analysis, but residual confounding might still exist.

In conclusion, we confirm earlier associations between iron status and cadmium levels, extend these findings to a novel patient setting (ie, KTRs), and highlight a subgroup of iron-deficient KTRs, who might be particularly vulnerable to cadmium-induced nephrotoxicity.

## Supplementary Materials

Supplementary File (PDF) Fig S1; Item S1; Tables S1, S2.
