# Supplementary material for: Iron Deficiency, Cadmium Levels, and Kidney Transplant Outcomes in Prevalent Kidney Transplant Recipients
Source: Kidney Med. 2024 Dec 20;7(2):100942. doi: 10.1016/j.xkme.2024.100942 (PMC11761878; doi:10.1016/j.xkme.2024.100942)
Supplement: Supplementary File (PDF) — Fig S1; Item S1; Tables S1, S2. [file mmc1.pdf]

Figure S1. Kaplan Meier curves showing the incidence of graft failure per cadmium tertile

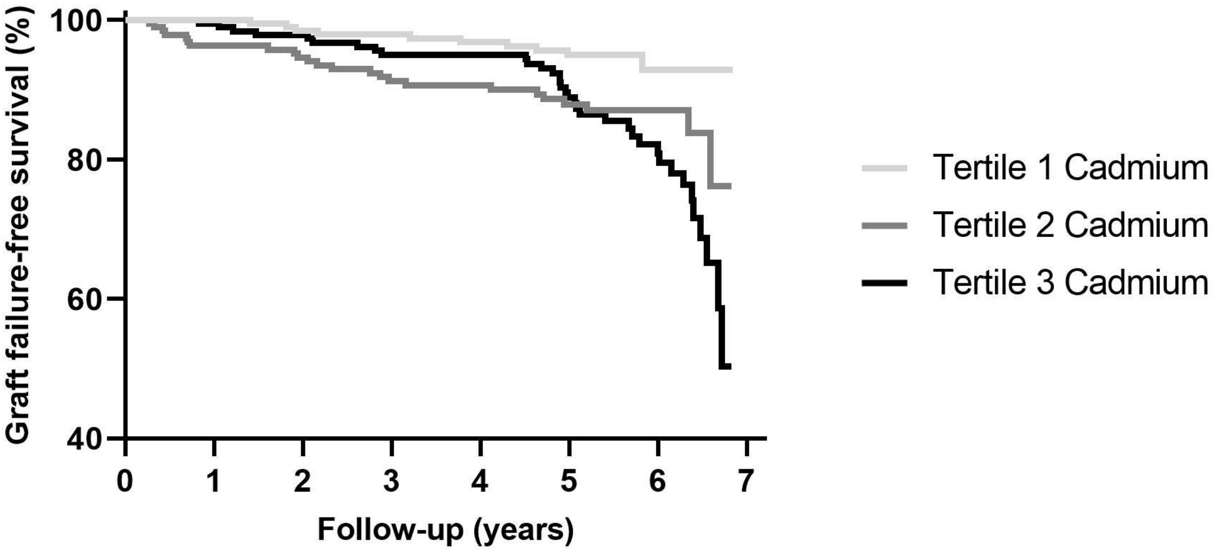

Kaplan-Meier Curve for death-censored graft failure (69 events in total).

Item S1: Supplementary Methods

Kidney transplant recipients (KTRs) ≥1 year after transplantation with available data regarding plasma cadmium levels, iron status, and kidney function parameters who participated in the TransplantLines Food and Nutrition Cohort study (Groningen, The Netherlands),[1] were included in the current study. All included participants signed informed consent and the study protocol has been approved by the institutional review board (METc 2008/186) and was conducted in accordance with the Declaration of Helsinki and Declaration of Istanbul. Plasma cadmium; transferrin saturation (TSAT), reflecting iron availability; and ferritin, reflecting iron storage, were measured at baseline. Kruskal-Wallis and Wilcoxon rank sum tests were used to compare plasma cadmium across tertiles of TSAT. Visual inspection of the data showed an inverse association of TSAT with log-transformed cadmium around the common iron deficiency cutoff value of TSAT 20%. Hence, the linear regression was estimated using natural splines with a knot placed at TSAT 20%. To investigate whether the association of plasma cadmium with graft failure differed depending on TSAT and ferritin levels, multivariable Cox regression analyses containing both main effects and their cross-product terms to test for interaction were used. A previous study suggested that the relationship between iron status and kidney function is non-linear in KTRs.[2] Therefore, it was decided to include tertiles of TSAT and ferritin levels with the middle tertile as the reference for the prospective graft failure analyses. The TSAT tertiles were: ≤20%, 21%-28%, and ≥29%. The ferritin tertiles were ≤72 µg/L, 73-174 µg/L, and ≥175 µg/L. Variables with a skewed distribution, i.e., cadmium and ferritin, were log-transformed for the linear regression analysis. A p-value <0.025 was considered significant (to correct for multiple testing) for all analyses except for interaction analyses. There, the significance level was set at <0.05.

Table S1. Baseline characteristics of kidney transplant recipients across tertiles of TSAT

		**TSAT**			
	**Total population (n= 574)**	**1st Tertile (≤20%) (n=192)**	**2nd Tertile (21-28%) (n=191)**	**3rd Tertile (≥29%) (n=191)**	***P*-value**
**Plasma cadmium (ng/dL)**	5.8 (4.4, 7.6)	6.2 (4.9, 7.7)	5.5 (4.1, 7.6)	5.6 (4.5, 7.3)	0.03
**Demographics**					
Age (yrs)	53 (12)	54 (12)	53 (13)	53 (12)	0.53
Sex (females, %)	242 (42%)	101 (53%)	76 (40%)	65 (34%)	<0.001
Weight (kg)	81 (17)	84 (17)	81 (18)	79 (14)	0.01
Waist (cm)	99 (15)	102 (15)	100 (14)	96 (14)	<0.001
Body mass index (kg/m2)	26.8 (4.9)	27.8 (5.2)	26.8 (5.1)	25.7 (4.0)	<0.001
Current smoker, n (%)	77 (13%)	18 (9.4%)	29 (15%)	30 (16%)	0.04
SBP (mmHg)	136 (17)	138 (18)	137 (19)	134 (15)	0.05
DBP (mmHg)	83 (11)	84 (11)	83 (11)	82 (11)	0.52
**Kidney function**					
eGFR (ml/min/1.73 m2)	52 (20)	51 (20)	52 (20)	52 (19)	0.43
Creatinine (µmol/L)	124 (99, 158)	126 (99, 163)	123 (98, 158)	123 (101, 153)	0.96
Urinary protein excretion (g/24 h)	0.19 (0.00, 0.34)	0.21 (0.00, 0.48)	0.19 (0.00, 0.32)	0.16 (0.00, 0.29)	0.03
Urinary albumin excretion (mg/24 h)	37 (10, 145)	52 (12, 205)	30 (10, 129)	31 (8, 119)	0.41
**Transplantation**					
Transplant vintage (yrs)	5 (2, 11)	4 (1, 10)	6 (3, 11)	6 (3, 14)	<0.001
Living donor, n (%)	198 (34%)	62 (32%)	73 (38%)	63 (33%)	0.41
Pre-emptive transplant, n (%)	95 (17%)	27 (14%)	40 (21%)	28 (15%)	0.13
Previous dialysis duration (months)	23 (4, 46)	25 (4, 47)	23 (0, 46)	20 (6, 47)	0.93
Cold ischemia times (minutes)	904 (169, 1,260)	908 (172, 1,231)	828 (160, 1,260)	960 (178, 1,299)	0.39
HLA mismatches, n	3(2,4)	3(2,4)	3(2,4)	3(2,4)	0.27
Acute rejection, n (%)	155 (27%)	51 (27%)	54 (28%)	50 (26%)	0.89
**Primary renal disease, n (%)**					0.25
Primary glomerular disease	172 (30%)	61 (32%)	52 (27%)	59 (31%)	
Glomerulonephritis	44 (7.7%)	14 (7.3%)	20 (10%)	10 (5.2%)	
Tubulointerstitial disease	57 (9.9%)	15 (7.8%)	18 (9.4%)	24 (13%)	
Polycystic renal disease	125 (22%)	45 (23%)	44 (23%)	36 (19%)	
Dysplasia and hypoplasia	19 (3.3%)	5 (2.6%)	4 (2.1%)	10 (5.2%)	

Renovascular disease	31 (5.4%)	12 (6.3%)	5 (2.6%)	14 (7.3%)	
Diabetic nephropathy	29 (5.1%)	11 (5.7%)	10 (5.2%)	8 (4.2%)	
Other or unknown cause	97 (17%)	29 (15%)	38 (20%)	30 (16%)	
**RBC and iron parameters**					
Hemoglobin (g/dL)	13.2 (1.7)	12.6 (1.7)	13.4 (1.7)	13.6 (1.6)	<0.001
TSAT (%)	25 (11)	14 (4)	24 (3)	37 (7)	<0.001
Ferritin (µg/L)	114 (53, 214)	48 (27, 110)	119 (72, 201)	180 (104, 292)	<0.001
**Inflammation**					
CRP (mg/L)	1.6 (0.8, 4.5)	2.5 (1.1, 6.7)	1.4 (0.7, 4.2)	1.2 (0.6, 2.8)	<0.001
Serum total protein (g/L)	71.1 (5.1)	71.2 (5.4)	71.4 (5.0)	70.9 (4.8)	0.72
**Other laboratory measurements**					
Cholesterol (mmol/L)	5.13 (1.12)	5.15 (1.13)	5.17 (1.18)	5.05 (1.06)	0.72
Phosphate (mmol/L)	0.97 (0.21)	0.98 (0.23)	0.97 (0.21)	0.95 (0.20)	0.68
Calcium (mmol/L)	2.40 (0.15)	2.38 (0.15)	2.42 (0.14)	2.39 (0.15)	0.08
PTH (pmol/L)	14 (30)	15 (35)	14 (15)	14 (36)	0.26

Normally distributed variables are reported as mean (SD) and skewed variables are reported as median (IQR). Abbreviations: CRP, C-reactive protein; DBP, diastolic blood pressure; eGFR, estimated glomerular filtration rate; HLA, human leukocyte antigen; PTH, parathyroid hormone; RBC, red blood cell; SBP, systolic blood pressure; TSAT, transferrin saturation.

Table S2. Baseline characteristics of kidney transplant recipients across tertiles of cadmium

		**Cadmium**			
	**Total population (n= 574)**	**1st Tertile (2.2-4.9 ng/dL) (n=192)**	**2nd Tertile (4.9-6.9 ng/dL) (n=191)**	**3rd Tertile (6.9-33.3 ng/dL) (n=191)**	***P*-value**
**Demographics**					
Age (yrs)	53 (12)	50 (13)	54 (12)	56 (11)	<0.001
Sex (females, %)	242 (42%)	66 (34%)	81 (42%)	95 (50%)	0.01
Weight (kg)	81 (17)	82 (16)	82 (17)	80 (17)	0.22
Waist (cm)	99 (15)	99 (13)	100 (16)	99 (15)	0.91
Body mass index (kg/m2)	26.8 (4.9)	26.5 (4.7)	27.2 (5.1)	26.6 (4.8)	0.51
Current smoker, n (%)	77 (13%)	21 (11%)	26 (14%)	30 (16%)	0.16
SBP (mmHg)	136 (17)	135 (17)	136 (16)	138 (19)	0.35
DBP (mmHg)	83 (11)	83 (11)	83 (11)	82 (11)	0.72
**Kidney function**					
eGFR (ml/min/1.73 m2)	52 (20)	59 (19)	51 (19)	45 (20)	<0.001
Creatinine (µmol/L)	124 (99, 158)	116 (97, 150)	125 (105,153)	141 (108,184)	<0.001
Urinary protein excretion (g/24 h)	0.19 (0.00, 0.34)	0.16 (0.00, 0.28)	0.19 (0.00, 0.30)	0.21 (0.00, 0.40)	0.11
Urinary albumin excretion (mg/24 h)	37 (10, 145)	28 (8, 121)	31 (12, 128)	55 (11, 180)	0.24
**Transplantation**					
Transplant vintage (yrs)	5 (2, 11)	7 (3, 12)	5 (1, 12)	5 (1, 10)	0.01
Living donor, n (%)	198 (34%)	77 (40%)	63 (33%)	58 (30%)	0.12
Pre-emptive transplant, n (%)	95 (17%)	40 (21%)	27 (14%)	28 (15%)	0.15
Previous dialysis duration (months)	23 (4, 46)	18 (0, 38)	23 (6, 46)	26 (6, 50)	0.03
Cold ischemia times (minutes)	904 (169, 1,260)	840 (155, 1,260)	960 (168, 1,286)	899 (180, 1,256)	0.36
HLA mismatches, n	3 (2,4)	3 (2,4)	3 (2,4)	3 (2,4)	0.54
Acute rejection, n (%)	155 (27%)	43 (22%)	58 (30%)	54 (28%)	0.19
**Primary renal disease, n (%)**					0.15
Primary glomerular disease	172 (30%)	67 (35%)	53 (28%)	52 (27%)	
Glomerulonephritis	44 (7.7%)	17 (8.9%)	16 (8.4%)	11 (5.8%)	
Tubulointerstitial disease	57 (9.9%)	22 (11%)	15 (7.9%)	20 (10%)	
Polycystic renal disease	125 (22%)	34 (18%)	45 (24%)	46 (24%)	
Dysplasia and hypoplasia	19 (3.3%)	7 (3.6%)	9 (4.7%)	3 (1.6%)	
Renovascular disease	31 (5.4%)	4 (2.1%)	13 (6.8%)	14 (7.3%)	
Diabetic nephropathy	29 (5.1%)	7 (3.6%)	8 (4.2%)	14 (7.3%)	

Other or unknown cause	97 (17%)	34 (18%)	32 (17%)	31 (16%)	
**RBC and iron parameters**					
Hemoglobin (g/dL)	13.2 (1.7)	13.7 (1.6)	13.2 (1.7)	12.8 (1.8)	<0.001
TSAT (%)	25 (11)	26 (9)	25 (11)	24 (11)	0.03
Ferritin (µg/L)	114 (53, 214)	125 (63, 197)	120 (50, 235)	105 (52, 204)	0.79
**Inflammation**					
CRP (mg/L)	1.6 (0.8, 4.5)	1.3 (0.6, 3.1)	1.7 (0.7, 5.0)	1.9 (0.9, 5.1)	0.01
Serum total protein (g/L)	71.1 (5.1)	70.8 (4.5)	71.6 (5.2)	71.0 (5.4)	0.10
**Other laboratory measurements**					
Cholesterol (mmol/L)	5.13 (1.12)	4.91 (1.02)	5.16 (1.12)	5.32 (1.19)	<0.001
Phosphate (mmol/L)	0.97 (0.21)	0.93 (0.21)	0.96 (0.21)	1.01 (0.21)	<0.001
Calcium (mmol/L)	2.40 (0.15)	2.39 (0.13)	2.40 (0.16)	2.40 (0.15)	0.77
PTH (pmol/L)	14 (30)	11 (10)	16 (47)	15 (20)	0.19

Normally distributed variables are reported as mean (SD) and skewed variables are reported as median (IQR). Abbreviations: CRP, C-reactive protein; DBP, diastolic blood pressure; eGFR, estimated glomerular filtration rate; HLA, human leukocyte antigen; PTH, parathyroid hormone; RBC: red blood cell; SBP, systolic blood pressure; TSAT, transferrin saturation.

Table S3. Cox regression for graft failure testing the interaction of cadmium with TSAT and ferritin

Models without interaction				Interaction models			
	**HR**	**95% CI**	**p-value**		**HR**	**95% CI**	**p-value**
Cadmium	1.09	1.02, 1.17	0.02	Cadmium * Low TSAT	1.21	1.01, 1.44	0.04
Low TSAT	1.69	0.87, 3.28	0.11	Cadmium * High TSAT	1.18	0.97, 1.44	0.10
High TSAT	1.28	0.69, 2.38	0.43	Cadmium * Low ferritin	0.96	0.79, 1.23	0.87
Low ferritin	0.54	0.27, 1.08	0.08	Cadmium * High ferritin	1.11	0.97, 1.25	0.15
High ferritin	1.21	0.68, 2.15	0.51				

Cox proportional hazard model with graft failure (median follow-up 5.6 years) as the outcome. Models are corrected for age, sex, smoking behavior, body mass index, estimated glomerular filtration rate, 24-hour urinary albumin excretion, time since transplantation, human leukocyte antigen mismatches, donor type, and clinical history of acute rejection. The middle tertile was regarded as the reference category. Abbreviations: CI, confidence interval; HR, hazard ratio; TSAT, transferrin saturation.

Supplementary References

1. Eisenga MF, Gomes-Neto AW, Van Londen M, et al. Rationale and design of TransplantLines: a prospective cohort study and biobank of solid organ transplant recipients. *BMJ Open*. 2018;8(12):e024502. doi:10.1136/BMJOPEN-2018-024502

2. Kim HJ, Han R, Kang KP, et al. Impact of iron status on kidney outcomes in kidney transplant recipients. *Sci Rep*. 2023;13(1). doi:10.1038/S41598-023-28125-X
